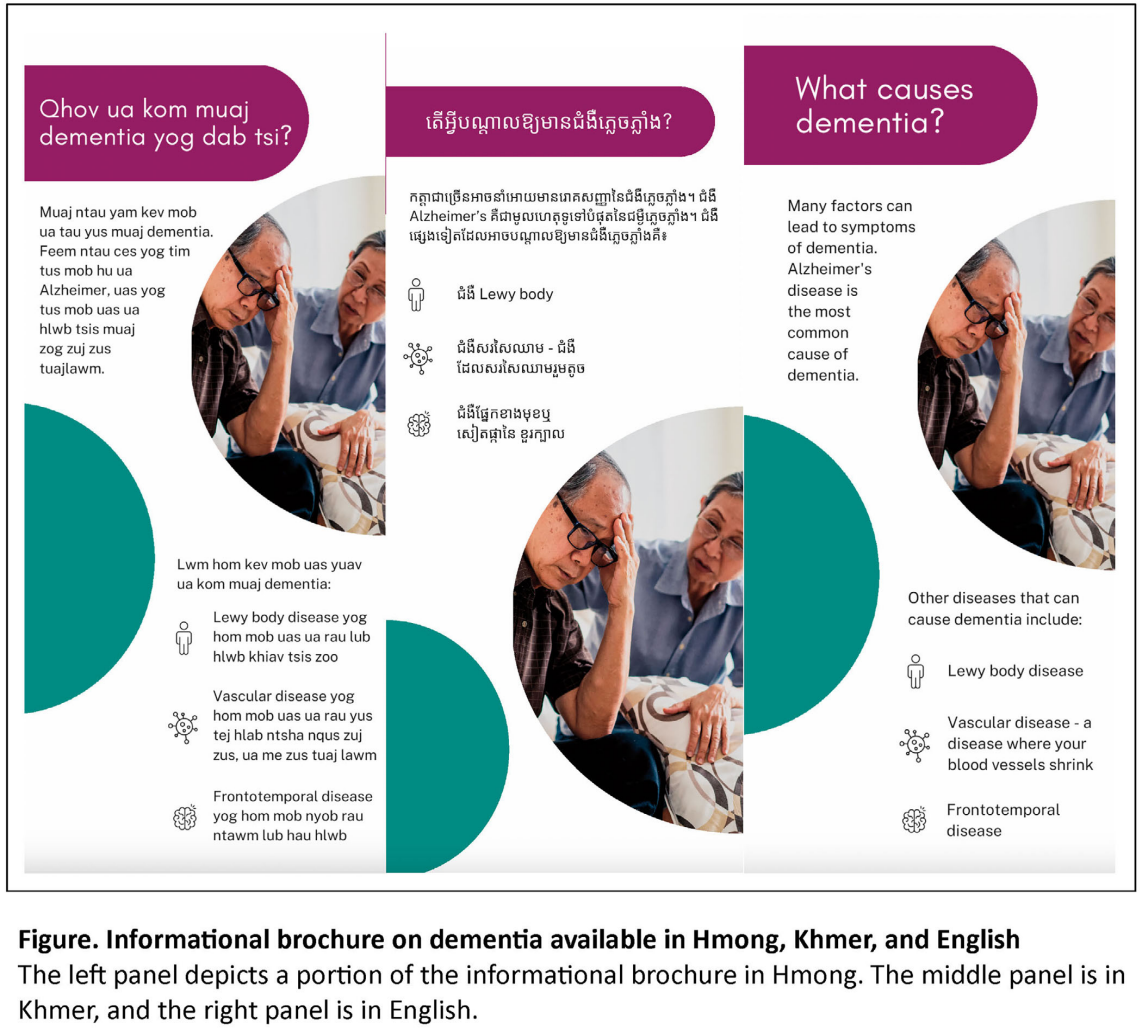# The process of creating culturally and linguistically appropriate materials on dementia for Hmong and Cambodian elders

**DOI:** 10.1002/alz.092766

**Published:** 2025-01-09

**Authors:** Kao Lee Yang, Yer Thor, Barbara B. Bendlin, Nathaniel A. Chin

**Affiliations:** ^1^ Neuroscience and Public Policy Program, University of Wisconsin School of Medicine and Public Health, Madison, WI USA; ^2^ Wisconsin Alzheimer’s Disease Research Center, University of Wisconsin School of Medicine and Public Health, Madison, WI USA; ^3^ Wisconsin Alzheimer’s Institute, University of Wisconsin School of Medicine and Public Health, Madison, WI USA; ^4^ VA Geriatric Research, Education and Clinical Center (GRECC), William S. Middleton Memorial Veterans Hospital, Madison, WI USA; ^5^ Wisconsin Alzheimer’s Institute, University of Wisconsin‐Madison School of Medicine and Public Health, Madison, WI USA; ^6^ Wisconsin Alzheimer’s Disease Research Center, Madison, WI USA

## Abstract

Hmong and Cambodian Americans, minoritized Asian American subgroups, are underrepresented in research, and prevalence of Alzheimer’s disease (AD) in these communities is unknown. However, our community partners in the Madison, Wisconsin area, who have served Hmong and Cambodian elders in the community for over 20 years, informed us in the fall of 2021 that they have encountered cases of dementia among elders they serve. They expressed a need for educational materials on dementia that could aid their community work. Following this meeting, we spent the year 2022‐2023 developing culturally and linguistically appropriate informational brochures on dementia.

Brochures were developed with funding from the UW‐Madison Morgridge Center for Public Service. The project involved a diverse team of dementia researchers, community partners with expertise in providing wraparound services to the Hmong and Cambodian communities, a geriatrician, professional Hmong and Cambodian translators, and a professional graphic designer. The brochure development process was iterative. We started by researching existing educational materials on dementia for individuals with various health literacies. A draft of a 3‐panel brochure was drawn up by the first author in English and discussed with the team. Verbiage for each panel was carefully chosen based on feedback from our community partners. Specifically, the brochure would minimize verbosity and technical terms so elders could more easily grasp concepts. The graphic designer skillfully enhanced our preliminary brochure design. The translators worked in consultation with the first author to ensure the brochure’s overall concepts were accurately translated. The final product was a single brochure on dementia available in Hmong, Khmer (the Cambodian language), or English (**Figure**). Brochures were then disseminated to our community partners and during community events in the spring and fall of 2023.

Our project demonstrates the feasibility of creating brochures that are culturally and linguistically tailored for the Hmong and Cambodian communities. We found the collaborative efforts of experts across various fields and vital insights from community partners familiar with target audiences to be imperative in our success. Similar initiatives should prioritize assembling diverse teams and engaging with community specialists at the beginning of the project to best reach underrepresented groups.